# Cold hardiness of *Corythucha marmorata* (Hemiptera: Tingidae) on the functional crop *Helianthus tuberosus*

**DOI:** 10.1038/s41598-025-95657-9

**Published:** 2025-04-02

**Authors:** Wei Zhou, Meng-Shuang Yao, Chang-Hao Lu, Hao-Jun Li, Wen-Long Chen

**Affiliations:** Guizhou Provincial Key Laboratory for Agricultural Pest Management of the Mountainous Region, Scientific Observing and Experimental Station of Crop Pest in Guiyang of Ministry of Agricultural and Rural Affairs, Institute of Entomology of Guizhou University, Guiyang, 550025 China

**Keywords:** Invasive species, *Corythucha marmorata*, Adult, Cold tolerance, *Helianthus tuberosus*, Invasive species, Entomology

## Abstract

The invasive phytophagous lace bug, *Corythucha marmorata*, threatens the functional food crop *Helianthus tuberosus*, but its overwintering ecology on this plant is poorly understood. This study evaluated the cold hardiness of *C. marmorata* at various life stages, focusing on the differences between female and male adults. *C. marmorata* overwinter as adults on *H. tuberosus*, based on a four-year winter field investigation. The supercooling and equilibrium freezing points of *C. marmorata* decline with development. Female adults showed the greatest supercooling capacity. The lower lethal temperature (female − 15 °C, male − 16 °C) is above the supercooling point (− 26 °C). The low temperature exposure mortality of *C. marmorata* female and male adults exhibited different regularities. We conclude that *C. marmorata* belongs to chill susceptible insects. October to February is the most recommended period for *C. marmorata* control by harvesting *H. tuberosus*. Weed removal, such as *Erigeron bonariensis*, *Erigeron canadensis*, and *Ambrosia trifida*, is an early control measure. These results enhance our understanding of *C. marmorata*’s cold tolerance and inform targeted pest management strategies for *H. tuberosus* crops.

## Introduction

*Helianthus tuberosus* (Angiospermae: Asteraceae), commonly referred to as Jerusalem artichoke, is cultivated for its tuber biomass^1^, utilized as a vegetable^2^, fodder crop^3^, and bioenergy source^4,5^ due to its elevated inulin content^6^. *H. tuberosus* can be cultivated in various growth conditions throughout multiple countries^7–9^. Functional dietary ingredients like it can improve health and reduce the risk of osteoporosis, colon cancer, and cardiovascular disease^10–12^. It is generally believed that *H. tuberosus* flourishes in nutrient-poor soil and demonstrates resilience to pests and diseases^13–15^. Nonetheless, this deficiency in knowledge may lead to insufficient pest control strategies for *H. tuberosus*.

In China, *Corythucha marmorata* (Hemiptera: Tingidae) is an invasive leaf-sucking pest that poses a significant threat to *H. tuberosus* and exhibits a wide range of host plants^16,17^. The host plants associated with *C. marmorata* belong to seven genera including of *Helianthus*^18,19^. The life cycle of *C. marmorata* comprises three stages: egg, nymph, and adult^20^. The nymph stage typically requires five instars prior to attaining sexual maturity in adulthood^21,22^. Both nymphs and adults of *C. marmorata* exhibit analogous feeding behaviors^23^. The feeding activity of nymphs and adults primarily occurs on the undersides of leaves, where they extract cellular contents from the upper palisade parenchyma layer, resulting in symptoms of chlorosis and stippling. In instances of severe infestation, newly emerging leaves may exhibit a bronzed or bleached appearance, and premature leaf abscission may take place^24^. When sweet potatoes *Ipomoea batatas* are unavailable in winter, *C. marmorata* is compelled to overwinter on the goldenrod *Solidago altissima* in Japan^18^. However, records of overwintering host plants for *C. marmorata* in China are limited. We hypothesize that *C. marmorata* may also migrate to other perennial plants during winter, such as *H. tuberosus*.

Winter in temperate regions presents significant challenges for insects. They encounter a range of abiotic and biotic stressors that function synergistically, and aligning life histories with the timing of seasonal shifts is essential for survival^25,26^. Insects, particularly invasive species, have evolved various strategies to survive at low temperatures in response to diverse climate change effects^27,28^. Whether invasive species can complete a generation to a new environment is determined by the temperature^29,30^. Comprehending the overwintering biology of invasive pests is essential for formulating effective control methods.

Cold hardiness, or cold tolerance, denotes the inherent ability of insects to endure both brief and prolonged exposure to low temperatures^25,31,32^. This characteristic, shaped by seasonal variations, geographical factors, host vegetation, developmental phase, and the intensity and length of cold exposure, is vital for insect survival, growth, reproduction, and notably, distribution^33,34^. The supercooling point (SCP) is the temperature at which spontaneous nucleation occurs, whereas the equilibrium freezing point (FP), similar to the melting point, is the temperature at which the final minute ice crystal evaporates when a frozen solution is gradually heated^35^. The equilibrium freezing point (FP) minus the supercooling point (SCP) is the supercooling capacity^35,36^. Insect responses to low temperatures are categorized into three categories: chill-susceptible (mortality primarily above SCP), freeze-avoidant (mortality primarily at SCP), and freeze-tolerant (mortality primarily below SCP)^27,34^. Moreover, winter surveys and subzero temperature exposure offer a direct means of studying habitats. For instance, analyzing the cold tolerance of insects can employ parameters such as semi-lethal temperature (LLT_50_), 99%-lethal temperature (LLT_99_), semi-lethal time (LLt_50_), and 99%-lethal time (LLt_99_) as critical indicators in the CLIMAX model to forecast their potential distributions, thereby improving the monitoring and management of populations^33,36,37^.

This study examined the cold adaptation of the invasive pest *C. marmorata* by analyzing winter population variations, measuring supercooling points and equilibrium freezing points, and assessing low temperature exposure. We discussed the impact of age and sex on supercooling capacity and predicted that *C. marmorata* may overwinter on *H. tuberosus*, with adult females exhibiting greater cold tolerance than adult males. Our objective was to focus on *C. marmorata*’s cold hardiness and propose environmentally sustainable pest management methods for *H. tuberosus*.

## Materials and methods

### Chrysanthemum lace bugs *Corythucha marmorata*

*C. marmorata* were collected from *H. tuberosus* plants located on the South Campus of Guizhou University (106°40′E, 26°25′N) in 2021. The lace bugs were maintained in an artificial chamber (Ningbo Jiangnan, Ningbo, China) with controlled temperature, humidity, and photoperiod conditions (26 °C, 14 h light/10 h dark photoperiod, and 65% ± 5% relative humidity). A circular piece of filter paper (8.8 cm in diameter) was moistened and affixed to a Petri dish (9 cm in diameter). Fresh *H. tuberosus* leaves were utilized to replace the old leaves, and the filter paper was replaced as necessary.

### Overwintering field survey

To investigate the overwintering habits of *C. marmorata*, a field survey was conducted in the Huaxi District of Guiyang City, Guizhou Province, during the winter months from November to January annually between 2020 and 2023. Based on prior observations^16^, two specific areas were selected as survey sites: the South Campus of Guizhou University (GZU, 106°40′E, 26°25′N) and the Teaching Experimental Farm of Guizhou University (TEF, 106°41′E, 26°24′N). These locations had not been subjected to pesticide treatments, and significant damage to *C. marmorata* was noted during the summer months. The survey was conducted through random sampling in green spaces adjacent to campus playgrounds, classrooms, dormitories, sidewalks, and agricultural lands surrounding the aforementioned sites. A five-point sampling method was employed to select 100 leaves from each location, which contained a variety of plant species. The host plants, along with their respective latitude and longitude coordinates, were tentatively recorded using a mark and recapture technique. During the winter season, seven species from the Asteraceae family were examined: *Erigeron canadensis*, *Helianthus tuberosus*, *Ambrosia trifida*, *Tagetes erecta*, *Erigeron bonariensis*, *Erigeron sumatrensis*, and *Crassocephalum crepidioides*. To assess the sex and survival status of the chrysanthemum lace bugs, leaves infested with these insects were collected and placed into a transparent insect box (20 cm × 12 cm × 8 cm). The sex of all collected specimens was accurately identified using a laboratory microscope (SN: 1J07758, SZ2-ILST, Olympus, Tokyo, Japan).

### The supercooling point and equilibrium freezing point measurements

**Fig. 1:**
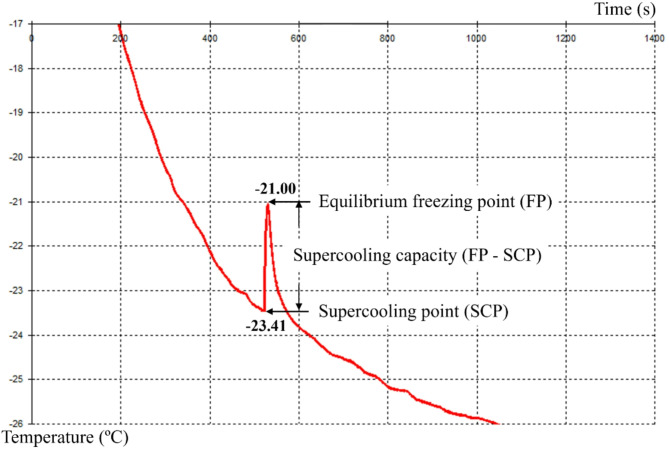
The display process of supercooling point and freezing point. *Corythucha marmorata* were affixed to a heat-sensitive probe, covered with defatted cotton, and placed in an ultralow temperature refrigerator set to − 40 °C. The insects’ body temperature initially drops to the supercooling point (SCP), where ice crystals begin to form. The release of latent heat then causes a rapid temperature increase until the ice crystals completely encase the insect, after which the temperature declines to the equilibrium freezing point (FP). The supercooling capacity is the difference between the equilibrium freezing point and the supercooling point.

We investigated the effect of life stage on the supercooling capacity of *C. marmorata* by examining all life stages from egg to adult. The experiment took place from November 4th to December 25th, 2021, using eggs (< 24 h old), and nymphs (< 12 h old). Overwintering adults were collected from *H. tuberosus* plants located on Tongcheng Southern Road in the Guanshan Lake District of Guiyang City (106°39′E, 26°39′N). Temperature measurement used an automatic temperature recorder (SN: 2020007559, SUN-V, Beijing, China). Insects were affixed to a heat-sensitive probe, covered with defatted cotton, and placed in an ultralow temperature refrigerator set to − 40 °C. The insects’ body temperature initially drops to the supercooling point (SCP), where ice crystals begin to form. The release of latent heat then causes a rapid temperature increase until the ice crystals completely encase the insect, after which the temperature declines to the equilibrium freezing point (FP) (Fig. 1). The values of SCP and FP were displayed on the computer monitor at the same time^16,38^. The tested insects were affixed to a heat-sensitive probe using transparent tape. We assessed a total of 40 eggs, 40 first instar nymphs, 40 second instar nymphs, 80 third instar nymphs, 80 fourth instar nymphs, 80 fifth instar nymphs, 80 overwintering male adults, and 80 overwintering female adults.

### Subzero temperature exposure

Samples collected during the wintering phase were used for low-temperature exposure experiments. *C. marmorata* shows remarkable resilience to temperature stress, with survival rates exceeding 90% for both sexes at temperatures above 0 °C for 2 h (Zhou, personal observations). However, survival rates drop to 0% at temperatures between − 26 °C and − 17 °C, with − 26 °C being the lowest supercooling point observed (Table 1). This limits the feasibility of dose-response analyses in this range. To assess low-temperature tolerance, we tested seven temperatures below 0 °C (− 16 °C to − 10 °C, inclusive) across four distinct stress durations (2, 4, 8, and 12 h), resulting in 28 experimental conditions. After exposure, individuals recovered at 26 °C for 24 h, and mortality was subsequently recorded. Survival was quantified using the semi-lethal low temperature (LLT_50_), lethal low temperature (LLT_99_), semi-lethal time (LLt_50_), and lethal time (LLt_99_) metrics. Each treatment group consisted of 30 male and 30 female adult lace bugs, with three replicates for each exposure condition.

**Table 1:** Supercooling point and equilibrium freezing point of *Corythucha marmorata* in life stages.

Life stages	Supercooling point (°C)	Equilibrium freezing point (°C)	Supercooling capacity (°C)
Egg	−7.44 ± 0.64 A	−5.42 ± 0.75 a	2.02
1st instar nymph	−12.63 ± 0.94 B	−10.10 ± 0.84 b	2.53
2nd instar nymph	−16.20 ± 0.87 C	−13.28 ± 1.29 c	2.92
3rd instar nymph	−18.17 ± 0.68 D	−15.71 ± 0.64 d	2.46
4th instar nymph	−20.59 ± 1.04 E	−17.67 ± 0.63 e	2.92
5th instar nymph	−20.61 ± 0.60 E	−17.28 ± 0.86 e	3.33
Male adult	−24.08 ± 0.68 F	−20.98 ± 1.10 f	3.10
Female adult	−26.18 ± 0.89 G	−22.13 ± 0.88 g	4.05

Values of the supercooling point and equilibrium freezing point, mean ± SD. The supercooling capacity is the difference between the equilibrium freezing point and the supercooling point. Different capital letters in the supercooling point indicate significant differences. Different lowercase letters in the equilibrium freezing point indicate significant differences. The same letters indicate no statistical differences (one-way ANOVA followed by Tukey test, *p* < 0.05).

### Data analysis

Data were analyzed utilizing IBM SPSS Statistics version 27 (SPSS, Chicago, IL, USA). A one-way analysis of variance (ANOVA) assessed normally distributed SCP and FP data across various life stages, confirmed by the Shapiro–Wilk test (*p* > 0.05). ANOVA identified significant disparities among stages, with a Tukey test for multiple comparisons. The mortality rate of *C. marmorata* after low temperature exposure was calculated as the ratio of deceased insects to the total tested, using both one-way ANOVA and probit regression. A *t*-test (*p* < 0.05) compared SCP/FP values between two nymphal stages and the mortality rates between female and male adults. Graphical representations were created with Prism 8.2.1 software (GraphPad Software, Inc., San Diego, CA, USA).

## Results

### Changes in adult frequency

**Fig. 2:**
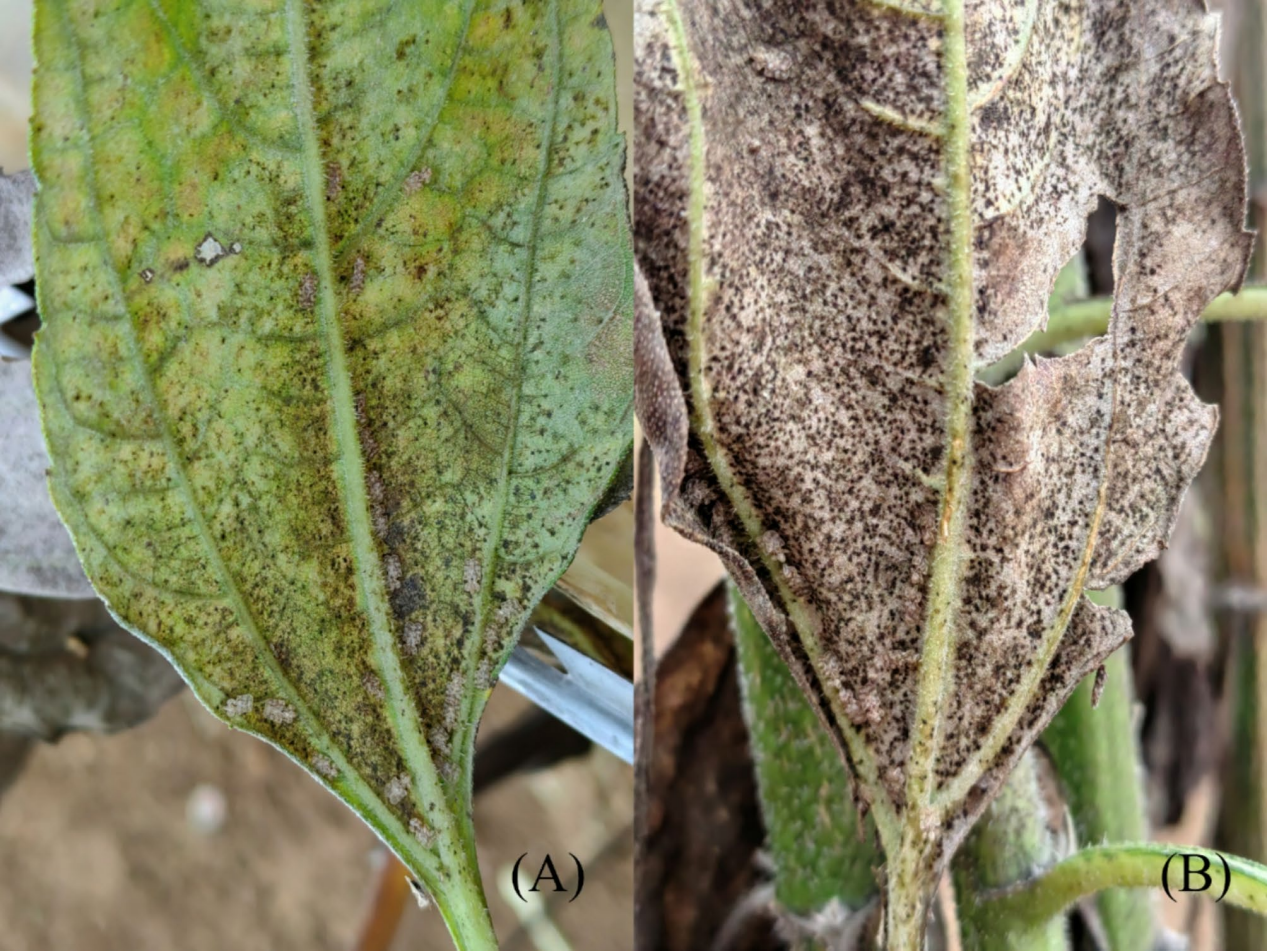
Damaged leaves of *Helianthus tuberosus* alongside overwintering adults of *Corythucha marmorata*. These photographs were captured by the authors on November 14th, 2023, within the Guanshan Lake District of Guiyang City, Guizhou Province, China (106°39′E, 26°39′N). In Panel A, the overwintering lace bugs congregate at the rear of the *H. tuberosus* leaves, their presence marked by punctate stripes against the foliage. Panel B reveals the remarkable camouflage of *C. marmorata*, whose body color blends seamlessly with the leaf’s hue.

Over four years of overwintering surveys, adults of *C. marmorata* specimens, including both females and males, were collected from two locations in Guiyang (Table 2). As of October 14, 2023, a total of 1,880 lace bugs were were gathered from various host plants. The highest adult frequency was recorded on *H. tuberosus* in December compared with that in November and January. It further indicates that *H. tuberosus* serves as the primary host for *C. marmorata*. The preferred overwintering sites were the undersides of leaves and the bases of stems, with a higher frequency of lace bugs found on leaves. *H. tuberosus* leaves displayed yellow and brown feeding damage caused by chrysanthemum lace bugs on the underside (Fig. 2A). Their brown mottled appearance made them hard to distinguish from the damaged leaves. Lace bugs were also found on withered leaves after segments of the plant had died (Fig. 2B).

**Table 2:** Overwintering situation of *Corythucha marmorata* in Huaxi district of Guiyang City.

Survey Date	Survey Location	Host Plants	Overwintering Site	Sex and Counts
Jan. 28th, 2021	TEF	*Helianthus tuberosus*	blade back of host plant	F: 57 M: 51
Jan. 8th, 2022	TEF	*Helianthus tuberosus*	blade back of host plant	F: 92 M: 68
Dec. 20th, 2022	TEF	*Helianthus tuberosus*	blade back of host plant	F: 200 M:155
Jan. 8th, 2022	TEF	*Tagetes erecta*	petiole of host plant	F: 2 M: 0
Jan. 4th, 2023	TEF	*Erigeron bonariensis*	blade back of host plant	F: 6 M: 2
Nov. 16th, 2022	TEF	*Erigeron canadensis*	blade back of host plant	F: 97 M: 88
Jan. 4th, 2023	TEF	*Erigeron canadensis*	blade back of host plant	F: 18 M: 14
Nov. 10th, 2020	GZU	*Erigeron canadensis*	blade back of host plant	F: 30 M: 13
Dec. 15th, 2020	GZU	*Helianthus tuberosus*	petiole of host plant	F: 90 M: 88
Nov. 19th, 2023	GZU	*Helianthus tuberosus*	blade back of host plant	F: 104 M: 85
Dec. 14th, 2023	GZU	*Helianthus tuberosus*	blade back of host plant	F: 95 M: 72
Nov. 3rd, 2021	GZU	*Ambrosia trifida*	blade back of host plant	F: 40 M: 37
Dec. 7th, 2021	GZU	*Ambrosia trifida*	blade back of host plant	F: 67 M: 58
Nov. 19th, 2023	GZU	*Erigeron sumatrensis*	petiole of host plant	F: 30 M: 39
Dec. 14th, 2023	GZU	*Crassocephalum crepidioides*	petiole of host plant	F: 4 M: 3

GZU, South Campus of Guizhou University; TEF, Teaching Experimental Farm of Guizhou University; F, overwintering female adult *C. marmorata*; M, overwintering male adult *C. marmorata*.

### Supercooling point and equilibrium freezing point

A comprehensive analysis of 520 lace bugs spanning all life stages of *C. marmorata* was conducted to assess their supercooling point and equilibrium freezing point (Table 1). A marked decline in supercooling point was discernible as the insects progressed through their life cycle (one-way ANOVA followed by Tukey test, *F*_7,188_ = 3574.1, *p* < 0.001, *R =* 0.99). The supercooling point peaked at the egg stage (− 7.44 °C) and reached its nadir in adult females (− 26.18 °C) (*t* = − 120.2, *df* = 118, *p* < 0.001). Furthermore, the supercooling point of female adults was significantly lower than that of male adults (*t* = − 16.5, *df* = 158, *p* < 0.001).

A substantial reduction in the equilibrium freezing point was also observed across different developmental stages (one-way ANOVA followed by Tukey test, *F*_7,187_ = 2276.4, *p* < 0.001, *R =* 0.98). The equilibrium freezing point reached its highest value at the egg stage (− 5.42 °C) and its lowest value at the female adult stage was for female adults (− 22.13 °C) (*t* = − 98.5, *df* = 512, *p* < 0.001). When compared to male adults, female adults demonstrated a significantly lower equilibrium freezing point (*t* = − 8.3, *df* = 158, *p* < 0.001). In summary, both the supercooling point and the equilibrium freezing point were statistically lower in adults than in their immature counterparts. Notably, adults displayed the most pronounced disparity of supercooling capacity compared to all nymphal stages.

### Mortality rate under low temperature exposure

**Fig. 3:**
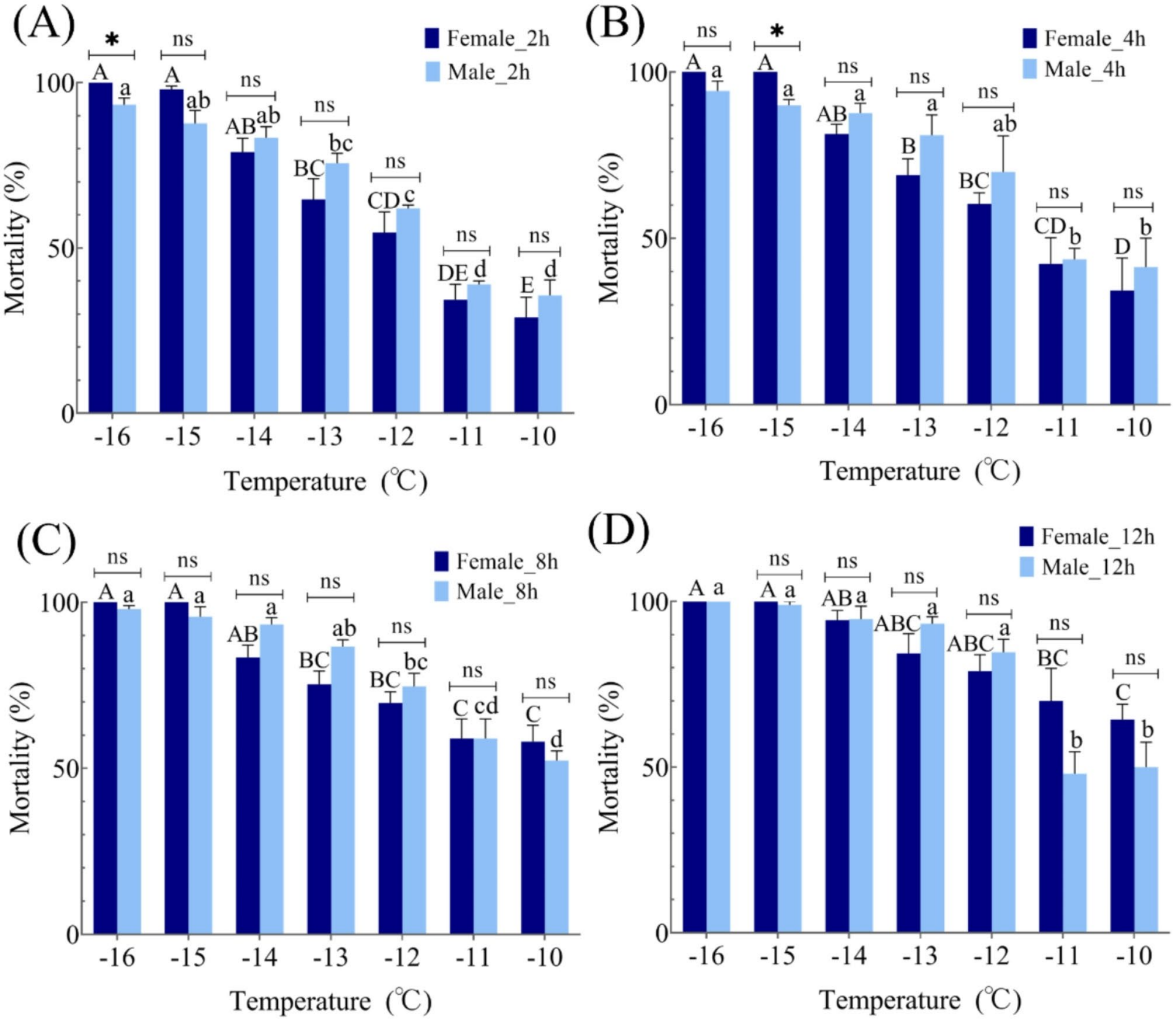
The mortality rates of *Corythucha marmorata* subjected to varying durations of low-temperature exposure. Panels A, B, C, and D represent exposures of 2 h, 4 h, 8 h, and 12 h, respectively. Significant differences among female adults are denoted by distinct capital letters, while those among male adults are indicated by different lowercase letters. The absence of statistical significance is marked by the use of identical letters (determined through one-way ANOVA, followed by Tukey’s test, with a significance threshold of *p* < 0.05). Additionally, asterisks (*) signify significant differences between female and male adults, whereas ‘ns’ denotes the absence of statistical differences between sexes (assessed via t-test, with a significance level of *p* < 0.05).

Cold exposure durations (2 h, 4 h, 8 h, 12 h) led to increased mortality rates in male and female *C. marmorata* adults as temperatures decreased (Fig. 3). Significant differences in mortality were noted at equivalent exposure durations (*p* < 0.001). After 2 h (Fig. 3A), most adults survived at − 10 °C and − 11 °C, with female mortality at 29–34.44% and male mortality at 35.67–39%. At − 12 °C, over half of the adults perished, with female mortality reaching 54.67% and male mortality surpassing 62%. Mortality rates stabilized from − 14 °C to − 16 °C, with females exceeding 79% and males surpassing 83.33%. A significant gender difference in mortality was observed at − 16 °C, with females at 100% and males at 93.33% (*t* = 3.288, *p* = 0.030).

After 4 h of exposure to cold temperatures (Fig. 3B), adult individuals could withstand temperatures as low as − 10 °C and − 11 °C. Female mortality rates ranged from 34.33 to 42.33%, while male rates were slightly higher, spanning from 41.33 to 43.67%. At − 13 °C, mortality rose sharply, with females at 69% and males at 81%. Female mortality plateaued above 81.33% below − 14 °C, while male mortality exceeded 70% below − 12 °C. Intriguingly, the only significant sex difference occurred at − 15 °C, where female mortality (100%) was notably higher than male mortality (90%) (*t* = 5.774, *p* = 0.004).

Exposure to cold temperatures of − 10 °C to − 16 °C for 8 h (Fig. 3C) resulted in 50% mortality among adult specimens, with female mortality exceeding 58% and male mortality surpassing 52.33%. Female mortality stabilized above 83.33% below − 14 °C, while male mortality plateaued at over 86.67% below − 13 °C. In an 8-hour exposure (Fig. 3D), over half of the adults died at all exposure temperatures, with female mortality surging significantly between − 10 °C and − 13 °C before stabilizing from − 14 °C to − 16 °C. Male mortality was similar at − 10 °C and − 11 °C, registering at 50% and 48%, respectively, before stabilizing between − 12 °C and − 16 °C. Furthermore, no significant difference in mortality rates was found between male and female adults in both 8-hour and 12-hour cold exposure scenarios.

### Lethal temperature under low temperature exposure

As illustrated in Table 3, the lethal temperature thresholds of *C. marmorata* under varying cold exposure durations were derived from regression analysis. The semi-lethal low temperature (LLT_50_) and lethal low temperature (LLT_99_) for adults increased with longer exposure. Notably, females exhibited higher lethal low temperatures than males at the same exposure duration (excluding 12 h). For females, LLT_50_ and LLT_99_ were − 12.923 °C and − 16.063 °C after 2 h, increasing slightly after 4 h (*p* < 0.001). After 8 h, LLT_50_ dropped to − 13.583 °C while LLT_99_ rose to − 15.482 °C (*p* < 0.042). After 12 h, both values stabilized at − 12.727 °C and − 15.372 °C, indicating a semi-lethal low temperature of approximately − 13 °C for female adults (*p* = 0.001). After 2 h of cold exposure, male adults had LLT_50_ and LLT_99_ values of − 11.278 °C and − 18.244 °C, respectively (*p* = 0.007). With increased exposure from 2 to 8 h, LLT_50_ and LLT_99_ rose by 0.589 °C and 1.553 °C (*p* = 0.045). After 12 h, LLT_50_ varied significantly at − 11.347 °C, while LLT_99_ increased to − 15.011 °C. Consequently, the semi-lethal low temperature for male adults is around − 11 °C (*p* < 0.001).

**Table 3:** Low lethal temperature of *Corythucha marmorata* exposed to different exposure durations.

Adult sex	Exposure durations (h)	Regression equation: y = k + Bx	*R* ^2^	LLT_50_ (°C)	95% Confidence interval	LLT_99_ (°C)	95% Confidence interval
Female	2	y = − 9.574 − 0.741x	0.861	−12.923	(− 13.345, − 12.239)	−16.063	(− 17.004, − 15.524)
	4	y = − 10.076 − 0.783x	0.773	−12.877	(− 13.440, − 11.616)	−15.850	(− 17.483, − 15.178)
	8	y = − 16.640 − 1.225x	0.676	−13.583	(− 14.038, − 5.499)	−15.482	(− 27.053, − 14.889)
	12	y = − 11.192 − 0.879x	0.567	−12.727	(− 13.321, − 11.063)	−15.372	(− 17.279, − 14.723)
Male	2	y = − 3.766 − 0.334x	0.915	−11.278	(− 13.170, − 6.057)	−18.244	(− 20.180, − 17.317)
	4	y = − 3.612 − 0.334x	0.739	−10.811	(− 13.326, − 0.359)	−17.775	(− 20.634, − 16.418)
	8	y = − 4.144 − 0.388x	0.890	−10.689	(− 13.007, − 0.676)	−16.691	(− 18.254, − 15.637)
	12	y = − 7.204 − 0.635x	0.760	−11.347	(− 12.510, − 7.097)	−15.011	(− 17.169, − 14.199)

*Corythucha marmorata* female and male adults were exposed to − 16 °C to − 10 °C. The regression equation was obtained by regression analysis, which was performed with the obtained data (repeated 90 times per treatment) at 2 h, 4 h, 8 h, and 12 h. In the regression equation, the letter x indicates exposure durations and the letter y indicates low temperature. *R*^2^, or the coefficient of determination, indicates the extent to which the independent variable x explains the variation in the dependent variable y.

### Lethal time under low temperature exposure

The experimental results showed that the low temperatures exposure mortality of *C. marmorata* had a certain regularity (Table 4). The semi-lethal time (LLt_50_) of female adults exposed to low temperatures (− 10 °C ~ − 14 °C) ranged from high (− 10 °C, 7.466 h) to low (− 12 °C, 0.360 h) then to high (− 14 °C, 10.433 h). The lethal time (LLt_99_) is from low (− 10 °C, 30.949 h) to high (− 12 °C, 34.128 h) then to low (− 14 °C, 16.587 h). At a certain temperature, all the females died. At − 15 °C and − 16 °C, all female adults died. For male adults, the low temperatures exposure mortality of *C. marmorata* had the different regularity (Table 4). The semi-lethal time (LLt_50_) of female adults exposed to low temperatures (− 10 °C ~ − 16 °C) ranged from high (− 10 °C, 9.942 h) to low (− 14 °C, 0.132 h) then to high (− 16 °C, 7.576 h). But the overall trend of the lethal time (LLt_99_) is from high (− 11 °C, 89.280 h) to low (− 16 °C, 10.375 h). Remarkably, some male adults survived at − 15 °C and − 16 °C. This study showed that *C. marmorata* responded to cold stress through the supercooling point and equilibrium freezing point, and male and female adults showed different cold tolerance.

**Table 4:** Low lethal time of *Corythucha marmorata* exposed to different exposure temperatures.

Adult sex	Exposure temperature (°C)	Regression equation: y = Intercept + Bx	*R* ^2^	LLt_50_ (h)	LLt_99_ (h)
Female	-10	y = − 0.740 + 0.099x	0.657	7.466	30.949
	-11	y = − 0.570 + 0.094x	0.622	6.084	30.932
	-12	y = − 0.025 + 0.069x	0.676	0.360	34.128
	-13	y = − 1.064 + 0.120x	0.503	8.895	28.336
	-14	y = − 3.944 + 0.378x	0.467	10.433	16.587
	-15	-	-	-	-
	-16	-	-	-	-
Male	-10	y = − 0.388 + 0.039x	0.343	9.942	69.608
	-11	y = − 0.258 + 0.029x	0.541	8.925	89.280
	-12	y = − 0.552 + 0.094x	0.407	5.872	30.619
	-13	y = − 0.335 + 0.112x	0.591	2.996	23.787
	-14	y = − 0.875 + 0.066x	0.345	0.132	21.889
	-15	y = − 1.280 + 0.229x	0.451	5.592	15.753
	-16	y = − 6.298 + 0.831x	0.311	7.576	10.375

*Corythucha marmorata* female and male adults were exposed to − 16 °C to − 10 °C. The survival rate (90 replicates per treatment) was recorded to calculate the regression equation of exposure temperature and semi-lethal time or lethal time. In the regression equation, the letter x indicates exposure temperature and the letter y indicates lethal time. *R*^2^, or the coefficient of determination, indicates the extent to which the independent variable x explains the variation in the dependent variable y.

## Discussion

Field surveys indicated that adults were present on *H. tuberosus* from November to January. Laboratory observations showed that *C. marmorata*, when fed *H. tuberosus*, experienced a decline in supercooling points and equilibrium freezing points as they matured. Females exhibited the highest supercooling capacity, while males survived temperatures as low as − 16 °C for nearly two hours. The lethal temperature is above the supercooling point (− 16 °C > − 26 °C), with females having a longer lethal time than males. According to the scheme of classification in the introduction, chill susceptible species are killed by cold in the absence of internal ice formation. Therefore, *C. marmorata* is considered chill-susceptible due to mortality occurring primarily above the supercooling point^39^. There are the following five aspects of *C. marmorata* on cold adaptation that need to be discussed.

First of all, *C. marmorata* overwinter as the adult form on *H. tuberosus* in all surveyed locations. The ratio of male to female population is close to 1:1. The findings of the overwintering form match those observed in previous studies. Many insects in the family Tingidae, such as *Corythucha ciliata*^40^, *Corythucha arcuata*^41^, and *Stephanitis nashi*^42^, all overwinter as adults. In the long-term evolutionary process, insects develop a fixed overwintering state^27^. In particular, *Stephanitis chinensis* overwinters in the egg stage^43^. We found that the supercooling points of *C. marmorata* eggs and nymphs are below a certain threshold (− 5 °C to − 20 °C), so they also exhibit a strong tolerance to low temperatures. The overwintering population numbers of females and males are close. This suggests that *C. marmorata* may have a robust reproductive foundation in winter. Since *C. marmorata* reproduces through male-female mating, it can maximize its population size when sufficient food sources become available in spring.

The second aspect involves the relationship between the supercooling point and cold tolerance. Our results show that the supercooling points and equilibrium freezing points decrease with development, consistent with findings for other insects of the same genus. For instance, the supercooling points of the oak lace bug *Corythucha arcuata* decreased from − 29.68 °C to −7.49 °C during overwintering^41^. Furthermore, the supercooling points of sycamore lace bug *Corythucha ciliata* female adults were lower than that of male adults under low temperatures, with − 11.49 °C and − 9.54 °C, respectively^40^. The measured supercooling point of *C. marmorata* (− 26.18 °C) was lower than that of *C. ciliata* (-11.49 °C)^40^, indicating that the northern boundary for overwintering may extend further north than that of *C. ciliata*. We speculate that other physiological responses also contribute to cold tolerance of *C. marmorata*. The acorn weevil, *Curculio glandium*, increased cold tolerance through fat and sugar accumulation, reduced water content, and increased bound water^44^. Additionally, the Antarctic collembolan, *Cryptopygus antarcticus*, increased cold tolerance through the hardening of the exoskeleton and removal of ice nuclei during molting, contributing to this effect^45^. The glycogen and lipid levels of woodboring pest, *Streltzoviella insularis*, also impact cold hardiness during overwintering^46^. Therefore, *C. marmorata* may adapt to low winter temperatures in these ways. A joint examination of the supercooling point of *C. marmorata* adults shows that it is the lowest, which verifies that *C. marmorata* primarily overwinters as adults, according to field surveys.

The third problem deals with ecological adaptation of *C. marmorata* under low temperature stress. The supercooling point is also influenced by ambient temperature, host plants, and season^37,46,47^. The supercooling point of the adult stage is lower than that of the other stages. Therefore, not all stages can survive in the winter. The overwintering behavior of lace bugs is closely linked to their location on the host plant. When exposed to low temperatures or freezing conditions during winter, these insects engage in specific behavioral activities to seek refuge. This adaptive mechanism is often referred to as an ecological adaptation strategy^48^. For instance, the overwintering sites of *C. ciliata* are primarily found in the bark of the trunk or the main branches of trees, allowing them to evade extreme cold. Additionally, the dried leaves of *H. tuberosus* provide a concealed overwintering habitat for *C. marmorata*, creating a microenvironment that offers some protection to the overwintering adults^49^.

In the fourth aspect, we evaluate the cold tolerance of *C. marmorata* by lethal temperature and lethal time. The comparison of lethal temperature thresholds between male and female adults exposed to low temperatures for 2 to 12 h revealed significant differences. *C. marmorata* responded to cold stress through the supercooling point and equilibrium freezing point, and male and female adults showed different cold tolerance. As the severity of low-temperature stress increases due to prolonged exposure and lower temperatures, male adults may exhibit a stronger cold tolerance than their female counterparts under the same low-temperature conditions. This may because that males tend to be smaller in size compared to females^22^ and possess a relatively reduced surface area to manage with exogenous low temperatures^31^. Meanwhile, the cold tolerance can be mainly improved through cold domestication including of rapid cold hardening and long-term cold acclimatization^27,50,51^. However, whether the differences in low-temperature survival rates between male and female individuals are a consistent phenomenon across the genus remains an area that warrants further investigation. To substantiate this hypothesis, it is imperative to conduct comprehensive ecological observations in conjunction with physiological and biochemical experiments.

On the last aspect, we indicate the corresponding measures of pest management according to the experimental results. Reports of economic crop destruction indicate a significant risk of pest invasion, with *C. marmorata* most frequently observed in December. Thus, October to February is the most recommended period for *C. marmorata* control. The widespread distribution of weed species necessitates their consideration in pest management. Both male and female adults are harmful to *H. tuberosus* and should be recognized as pests. Given the economic potential of *H. tuberosus*, urgent research on new variety breeding is needed to reduce *C. marmorata* damage. For example, a 10% acetamiprid microemulsion can be selected as the best control agent^52^. As a predatory natural enemy, *Stethoconus japonicas* has a good predation preference for five species of Tingidae, including of *C. marmorata*, thus achieving green prevention and control^53^. Clearing the source of pests is important. We found there were overwintering *C. marmorata* on weeds such as *Erigeron bonariensis*, *Erigeron canadensis*, and *Ambrosia trifida*, so it was necessary to remove deciduous leaves and weeds in the garden in a timely manner. Compared to chemical and biological control, direct weed removal can reduce the number of overwintering insects. At the same time, eradication of field weeds (*C. marmorata* host plants) in large-scale agricultural systems can prevent *C. marmorata* from being attracted to reproduce in the field and damage crops, thereby protecting crops such as *H. tuberosus*.

This study explores cold hardiness differences between female and male *C. marmorata*, with *H. tuberosus* identified as the main overwintering host plant. The supercooling capacity may vary with different host plants, suggesting that feeding lace bugs different plants could alter the supercooling capacity. Future research should expand winter investigations to clarify the physiological and molecular factors affecting the supercooling point and uncover low-temperature tolerance mechanisms in invasive species, enhancing our understanding of low-temperature biology.

## Conclusion

This research indicates that the damage frequency of *C. marmorata* on *H. tuberosus* was the highest, with the species primarily overwintering as adults. These overwintering adults predominantly inhabit the undersides of *H. tuberosus* leaves, resulting in extremely high population numbers of *C. marmorata*. The sex ratio of female to male adults is approximately 1:1. *C. marmorata* copes with low winter temperatures through the supercooling point and equilibrium freezing point. In certain regions, such as Guiyang, China, *C. marmorata* adults can survive on *H. tuberosus* throughout the winter. To reduce the cardinal number of overwintering insects, the lower leaves of *H. tuberosus* were mainly removed from October to February. Furthermore, further work is needed to clarify the factors influencing the supercooling point and to uncover the low-temperature tolerance mechanisms in invasive species, thereby enhancing our understanding of low-temperature biology.

## Data Availability

The data that support the findings of this study are available from [Guizhou University] but restrictions apply to the availability of these data, which were used under license for the current study, and so are not publicly available. Data are however available from the corresponding authors upon reasonable request and with permission of [Guizhou University].

## References

[1] Yang, L., He, Q. S., Corscadden, K. & Udenigwe, C. C. The prospects of Jerusalem artichoke in functional food ingredients and bioenergy production. *Biotechnol. Rep.* **5**, 77–88. 10.1016/j.btre.2014.12.004 (2015). 10.1016/j.btre.2014.12.004 PMC5466194 28626686

[2] Sawicka, B. & Krochmal-Marczak, B. *Jerusalem Artichoke Food Science and Technology: Helianthus Tuberosus*. 1–253 (Springer Nature, 2022).

[3] Cornescu, G. M., Panaite, T. D., Soica, C., Cismileanu, A. & Matache, C. C. Jerusalem artichoke (*Helianthus tuberosus* L.) as a promising dietary feed ingredient for monogastric farm animals. *Appl. Sci.* **13**, 12748. 10.3390/app132312748 (2023).

[4] Chen, Y. et al. Two-stage pretreatment of Jerusalem artichoke stalks with wastewater recycling and lignin recovery for the biorefinery of lignocellulosic biomass. *Processes* **11**, 127. 10.3390/pr11010127 (2023).

[5] Dalmis, R. Description of a new cellulosic natural fiber extracted from *Helianthus tuberosus* L. as a composite reinforcement material. *Physiol. Plant.* **175** (4), e13960. 10.1111/ppl.13960 (2023). 37339003 10.1111/ppl.13960

[6] Paul, J. D., Lutsiv, T. & Thompson, H. J. A perennial green revolution to address 21st-century food insecurity and malnutrition. *Food Energy Secur.* **13**, e568. 10.1002/fes3.568 (2024).

[7] Chaimala, A., Jogloy, S., Vorasoot, N., Holbrook, C. C. & Kvien, C. K. The roles of net photosynthetic rate and transpiration efficiency on economic yield of Jerusalem artichoke (*Helianthus tuberosus* L.) genotypes under different drought durations during the terminal growth stages. *Agronomy* **13**, 1882. 10.3390/agronomy13071882 (2023).

[8] Zhao, M., Ren, Y. & Li, Z. Transcriptome profiling of Jerusalem artichoke seedlings (*Helianthus tuberosus* L.) under polyethylene glycol-simulated drought stress. *Ind. Crops Prod.* **170**, 113696. 10.1016/j.indcrop.2021.113696 (2021). 10.3390/ijms22073294 PMC8037225 33804948

[9] Zou, H. X. et al. Salt stress induced differential metabolic responses in the sprouting tubers of Jerusalem artichoke (*Helianthus tuberosus* L). *PLoS One*. **15**, e0235415. 10.1371/journal.pone.0235415 (2020). 32598354 10.1371/journal.pone.0235415 PMC7323981

[10] Aliasgharzadeh, A. et al. A combination of prebiotic inulin and oligofructose improve some of cardiovascular disease risk factors in women with type 2 diabetes: A randomized controlled clinical trial. *Adv. Pharm. Bull.* **5**, 507–514. 10.15171/apb.2015.069 (2015). 26819923 10.15171/apb.2015.069 PMC4729356

[11] Nair, K. K., Kharb, S. & Thompkinson, D. K. Inulin dietary fiber with functional and health attributes—A review. *Food Rev. Int.* **26**, 189–203. 10.1080/87559121003590664 (2010).

[12] Long, X. H., Shao, H. B., Liu, L., Liu, L. P. & Liu, Z. P. Jerusalem artichoke: A sustainable biomass feedstock for biorefinery. *Renew. Sustain. Energy Rev.* **54**, 1382–1388. 10.1016/j.rser.2015.10.063 (2016).

[13] Bedzo, O. K. K., Mandegari, M. & Görgens, J. F. Techno-economic analysis of inulooligosaccharides, protein, and biofuel co-production from Jerusalem artichoke tubers: A biorefinery approach. *Biofuels Bioprod. Biorefin*. **14**, 776–793. 10.1002/bbb.2105 (2020).

[14] Bembenek, M. et al. Jerusalem artichoke as a Raw material for manufacturing alternative fuels for gasoline internal combustion engines. *Energies* **17**, 2378. 10.3390/en17102378 (2024).

[15] Ozgoren, E., Isik, F. & Yapar, A. Effect of Jerusalem artichoke (*Helianthus tuberosus* L.) supplementation on chemical and nutritional properties of crackers. *J. Food Meas. Charact.* **13**, 2812–2821. 10.1007/s11694-019-00201-9 (2019).

[16] Zhou, W. & Chen, W. L. Early starvation contributes to the adaptive capacity of *Corythucha marmorata* (Uhler), an emerging pest in China. *Biology* **11** (1), 80. 10.3390/biology11010080 (2022). 35053078 10.3390/biology11010080 PMC8772960

[17] Li, N. et al. Forecast of current and future distributions of *Corythucha marmorata* (Uhler) under climate change in China. *Forests* **15**, 843. 10.3390/f15050843 (2024).

[18] Rizkawati, V., Sakai, K., Tsuchiya, T. & Tsukada, M. Different egg size in the chrysanthemum lace bug *Corythucha marmorata* (Hemiptera: Tingidae) in response to novel host plant cultivars. *Appl. Entomol. Zool.* **58** (1), 93–103. 10.1007/s13355-022-00808-3 (2023).

[19] Sakata, Y. Geographic variation and Temporal changes in plant-herbivore interaction: case studies with *Solidago altissima* in native and introduced ranges. *Plant. Spec. Biol.* **37** (1), 6–19. 10.1111/1442-1984.12353 (2022).

[20] Bailey, N. S. The Tingoidea of new England and their biology. *Entomol. Am.* **31**, 1–140 (1951).

[21] Cappuccino, N. & Root, R. B. The significance of host patch edges to the colonization and development of *Corythucha marmorata* (Hemiptera: Tingidae). *Ecol. Entomol.* **17**, 109–113. 10.1111/j.1365-2311.1992.tb01166.x (1992).

[22] Shen, J. S., Zhu, M., Cui, X. H. & Li, L. J. Life table and biological characteristics of an exotic lace bug, *Corythucha marmorata* (Uhler). *Chin. J. Appl. Ecol.* **27** (5), 1657–1662. 10.13287/j.1001-9332.201605.027 (2016). 10.13287/j.1001-9332.201605.027 29732829

[23] Medina, L. S. & Craig, T. P. The impact of plant genetic variation and water stress on plant–herbivore interactions. *Ecol. Entomol.* **49**, 518–529. 10.1111/een.13324 (2024).

[24] Braman, S. K., Nair, S. & Carr, E. Influence of temperature, CO_2_ concentration, and species on survival and development of lace Bugs (Hemiptera: Tingidae). *J. Entomol. Sci.* **48**, 251–254. 10.18474/0749-8004-48.3.251 (2013).

[25] Denlinger, D. L. & Lee, J. R. E. *Low Temperature Biology of Insects*. 1–390 (Cambridge University Press, 2010).

[26] Teets, N. M., Marshall, K. E. & Reynolds, J. A. Molecular mechanisms of winter survival. *Annu. Rev. Entomol.* **68**, 319–339. 10.1146/annurev-ento-120120-095233 (2023). 36206770 10.1146/annurev-ento-120120-095233

[27] Bale, J. S. & Hayward, S. A. L. Insect overwintering in a changing climate. *J. Exp. Biol.* **213** (6), 980–994. 10.1242/jeb.037911 (2010). 20190123 10.1242/jeb.037911

[28] Ullah, F. et al. Insect resilience: unraveling responses and adaptations to cold temperatures. *J. Pest Sci.* **97**, 1153–1169. 10.1007/s10340-023-01741-2 (2024).

[29] Liu, X., Huang, W., Liu, Y. J. & Zhan, A. B. Perspectives of invasive alien species management in China. *Ecol. Appl.* **34** (1), e2926. 10.1002/eap.2926 (2024). 37864784 10.1002/eap.2926

[30] Paini, D. R. et al. Global threat to agriculture from invasive species. *Proc. Natl. Acad. Sci. U. S. A.* **113**(27), 7575–7579 (2016). 10.1073/pnas.1602205113 10.1073/pnas.1602205113 PMC4941431 27325781

[31] Lee, R. Insect cold-hardiness: to freeze or not to freeze. *BioScience* **39** (5), 308–313. 10.2307/1311113 (1989).

[32] Stephens, A. R., Asplen, M. K., Hutchison, W. D. & Venette, R. C. Cold hardiness of winter-acclimated *Drosophila Suzukii* (Diptera: Drosophilidae) adults. *Environ. Entomol.* **44**, 1619–1626. 10.1093/ee/nvv134 (2015). 26317777 10.1093/ee/nvv134

[33] Shi, F. et al. Unveiling winter survival strategies: physiological and metabolic responses to cold stress of *Monochamus saltuarius* larvae during overwintering. *Pest Manage. Sci.* **80** (11), 5656–5671. 10.1002/ps.8282 (2024). 10.1002/ps.8282 38979967

[34] Sinclair, B. J., Alvarado, C., Ferguson, L. V. & L. E. & An invitation to measure insect cold tolerance: methods, approaches, and workflow. *J. Therm. Biol.* **53**, 180–197. 10.1016/j.jtherbio.2015.11.003 (2015). 26590471 10.1016/j.jtherbio.2015.11.003

[35] Zachariassen, K. E. & Kristiansen, E. Ice nucleation and antinucleation in nature. *Cryobiology* **41**, 257–279. 10.1006/cryo.2000.2289 (2000). 11222024 10.1006/cryo.2000.2289

[36] Renault, D., Salin, C., Vannier, G. & Vernon, P. Survival at low temperatures in insects: what is the ecological significance of the supercooling point? *Cryoletters* **23**, 217–228 (2002). 12391482

[37] Kalushkov, P. & Nedvěd, O. Cold hardiness of *Pyrrhocoris apterus* (Heteroptera: Pyrrhocoridae) from central and Southern Europe. *Eur. J. Entomol.* **97**, 149–153. 10.14411/eje.2000.027 (2000).

[38] Chen, S. Y. et al. Cold tolerance strategy and cryoprotectants of *Megabruchidius dorsalis* in different temperature and time stresses. *Front. Physiol.* **13**, 1118955. 10.3389/fphys.2022.1118955 (2023). 36714316 10.3389/fphys.2022.1118955 PMC9873968

[39] Izadi, H., Cuthbert, R. N., Haubrock, P. J. & Renault, D. Advances in Understanding lepidoptera cold tolerance. *J. Therm. Biol.* **125**, 103992. 10.1016/j.jtherbio.2024.103992 (2024). 39418723 10.1016/j.jtherbio.2024.103992

[40] Ju, R. T., Wang, F., Xiao, Y. Y. & Li, B. Supercooling capacity and cold hardiness of the adults of the sycamore lace bug, *Corythucha Ciliata* (Hemiptera: Tingidae). *Cryoletters* **31**(6), 445–453 (2010). 21410013

[41] Paulin, M. J., Eötvös, C. B., Zabransky, P., Cso, G. & Schebeck, M. Cold tolerance of the invasive oak lace bug, *Corythucha arcuata*. *Agr For. Entomol.* **25** (4), 612–621. 10.1111/afe.12585 (2023).

[42] Wang, X., Zhang, X., Hu, C. & Sun, C. The insects of Tingidae from Jiangsu Province (Hemiptera: Tingoidea). *Jiangsu Agricultural Sci.* **41**, 124–126 (2013).

[43] Yin, Z. Y. et al. The Spatial distribution patterns of and sampling technique for *Stephanitis chinensis* Drake. *Plant. Prot.* **48** (4), 226–230 (2022).

[44] Udaka, H. & Sinclair, B. J. The overwintering biology of the acorn weevil, *Curculio Glandium* in Southwestern Ontario. *J. Therm. Biol.* **44**, 103–109. 10.1016/j.jtherbio.2014.02.019 (2014). 25086980 10.1016/j.jtherbio.2014.02.019

[45] Worland, M. & Convey, P. The significance of the moult cycle to cold tolerance in the Antarctic Collembolan *Cryptopygus antarcticus*. *J. Insect Physiol.* **54** (8), 1281–1285. 10.1016/j.jinsphys.2008.06.009 (2008). 18662695 10.1016/j.jinsphys.2008.06.009

[46] Pei, J. H., Li, C. C., Ren, L. L. & Zong, S. X. Factors influencing cold hardiness during overwintering of *Streltzoviella insularis* (Lepidoptera: Cossidae). *J. Econ. Entomol.* **113** (3), 1254–1261. 10.1093/jee/toaa032 (2020). 32161958 10.1093/jee/toaa032

[47] Ditrich, T. Supercooling point is an individually fixed metric of cold tolerance in *Pyrrhocoris apterus*. *J. Therm. Biol.* **74**, 208–213. 10.1016/j.jtherbio.2018.04.004 (2018). 29801629 10.1016/j.jtherbio.2018.04.004

[48] Li, N. G. Strong tolerance to freezing is a major survival strategy in insects inhabiting central Yakutia (Sakha Republic, Russia), the coldest region on Earth. *Cryobiology* **73** (2), 221–225. 10.1016/j.cryobiol.2016.07.007 (2016). 27424094 10.1016/j.cryobiol.2016.07.007

[49] Košťál, V. & Šimek, P. Overwintering strategy in *Pyrrhocoris apterus* (Heteroptera): the relations between life-cycle, chill tolerance and physiological adjustments. *J. Insect Physiol.* **46**, 1321–1329. 10.1016/S0022-1910(00)00056-1 (2000). 10844151 10.1016/S0022-1910(00)00056-1

[50] Teets, N. M., Gantz, J. D. & Kawarasaki, Y. Rapid cold hardening: ecological relevance, physiological mechanisms and new perspectives. *J. Exp. Biol.* **223**, jeb203448. 10.1242/jeb.203448 (2020). 32051174 10.1242/jeb.203448

[51] Hoffmann, A. A. Rapid adaptation of invertebrate pests to Climatic stress? *Curr. Opin. Insect Sci.* **21**, 7–13. 10.1016/j.cois.2017.04.009 (2017). 28822492 10.1016/j.cois.2017.04.009

[52] Wang, Z. H. et al. The toxicity and control efficacy of different pesticides on *Corythucha marmorata*. *Agrochemicals* **58** (2), 136–140 (2019).

[53] Luo, Y. et al. Predation on five species of Tingidae (Hemiptera) by *Stethoconus Japonicas*. *Chin. J. Appl. Entomol.* **57**, 413–420 (2020).

